# Downregulationof circ_0001578 promotes gestational diabetes mellitus by inducing placental inflammation *via* the NF-κB and JNKs pathways

**DOI:** 10.3389/fendo.2022.657802

**Published:** 2022-10-03

**Authors:** Wei Zhang, Xudong Zhao, Ling Li

**Affiliations:** ^1^ Department of Endocrinology, Shengjing Hospital of China Medical University, Shenyang, China; ^2^ Department of Otolaryngology Head and Neck Surgery, Shengjing Hospital of China Medical University, Shenyang, China

**Keywords:** circ_0001578, gestational diabetes mellitus, inflammation, NF-κB, JNKs, plasma exosome

## Abstract

Gestational diabetes mellitus (GDM) is one of the most common diseases during pregnancy. Some patients with GDM have adverse pregnancy outcomes. However, the pathogenesis of GDM is very complex and not well understood. In this study, we characterized the expression and functions of a circular RNA, circ_0001578, in GDM. In particular, using qRT-PCR, we verified previous RNA-seq results showing that circ_0001578 is significantly downregulated in the placental villous tissues of pregnant women with GMD. We demonstrated that plasma exosome circ_0001578 expression in the second trimester effectively predicts GDM at 28 weeks. Furthermore, in HTR-8/SVneo trophoblasts, the downregulation of circ_0001578 inhibited proliferation and migration and induced apoptosis. These changes may induce chronic inflammation in the placenta. These effects of circ_0001578 downregulation may be mediated by the upregulation of the NF-κB and JNK pathways, combined with increased expression levels of IL-1, IL-6, IL-8, TNF-α, and CRP. Collectively, the downregulation of circ_0001578 may promote GDM by inducing chronic inflammation in the placenta *via* the NF-κB and JNK pathways. Furthermore, our findings support that circ_0001578 has potential to serve as an early marker of GDM.

## Introduction

Gestational diabetes mellitus (GDM), which refers to any degree of glucose intolerance with first diagnosis or onset in pregnancy, is the most common disease during pregnancy. Most women with GDM have normal blood glucose levels after delivery. However, the risk of developing type II diabetes increases a few years later. GDM may cause many complications, such as macrosomia, stillbirth, and abortion, with adverse effects on pregnant women and fetuses (1, 2). In patients with GDM, the incidence rate of spontaneous abortion is 15%–30%, the rate of pregnancy-induced hypertension syndrome is 3 to 5 times that of normal pregnant women, the incidence of abnormal amniotic fluid is 13%–36%, and the incidence of macrosomia is 25%–40% (3). GDM also increases the incidence of infection, ketoacidosis, premature birth, intrauterine growth restriction, perinatal death, and fetal malformation (3). GDM also increases metabolic risks and complications during pregnancy for women and offspring (4, 5). Controlling blood glucose within a reasonable range can improve the prognosis (6). The incidence of GDM worldwide is increasing, affecting an estimated 20 million pregnant women every year (about one-sixth of pregnancies). In particular, 90% of cases occur in Southeast and South Asia, and 17.5% of pregnant women are affected by GDM in China (7). Diagnosis and medical intervention are essential for improving pregnancy outcomes in patients with GDM. However, the pathogenesis of GDM is very complex and the etiology of GDM is still elusive. The classical view is that placental hormones, prolactin, progesterone, and glucocorticoid antagonize the increased insulin level and insulin resistance during pregnancy is an important factor in the pathogenesis of GDM (8, 9). However, it is difficult to explain the mechanism underlying GDM in pregnant women with physiological insulin resistance. Recent studies have shown that some inflammatory factors, adipokines, glycosylated hemoglobin, and serum resistin play important roles in gestational insulin resistance, providing direction for further analyses of the pathogenesis of GDM (10–12).

Circular RNAs (circRNAs) are endogenous noncoding RNAs consisting of a circular configuration by typical 5′ to 3′-phosphodiester bonds (13–16). Compared with traditional linear RNAs, circRNAs have a more stable closed-loop structure. Previously, circRNAs have been recognized as the product of abnormal gene splicing, although there are a number of circRNAs within the human transcriptome (17, 18). CircRNAs contribute to many pathological and physiological processes *via* the transcriptional and posttranscriptional regulation of gene expression (19, 20). CircRNAs also play roles in many physiological processes by forming RNA–protein complexes to regulate transcription (21), encoding proteins (22), and sponging miRNAs to act as competitive endogenous RNAs (23).They have roles in maintaining the stability of mRNAs and sponging microRNAs and regulating RNA-binding proteins and translation (20, 21, 23). There is increasing evidence that circRNAs regulate cell functions and many human diseases, including GDM (24–31). Due to improvements in high-throughput sequencing technologies, increasing numbers of circRNAs have been reported to have regulatory functions in various diseases (32, 33). Recent research has shown that circRNAs play roles in the progression of many diseases, such as solid tumors (24–26), disorders of the nervous system (27, 28), atherosclerosis (29), and diabetes mellitus (30). Wang et al. found abnormally expressed circRNAs in placental villous tissues of patients with GDM by RNA sequencing (RNA-seq); in particular, circ_0001578 was significantly downregulated in the placental villous tissue of pregnant women with GMD (31).

GDM involves the abnormal function and inflammation of placental villous tissues (34). The placenta is responsible for the exchange of gases and nutrients between the mother and fetus. A series of changes occur in the placenta of patients with GDM, including inflammation and the differential expression of circRNAs (34, 35). However, little is known about the functions of circRNAs, including circ_0001578, in the abnormal function and inflammation of placental villous tissues in GDM.

In this study, we investigated the expression patterns and function of circRNA_0001578 in the placenta and the mechanisms underlying its functions. Our results clearly establish circRNA as a potential target for the diagnosis and treatment of GDM.]

## Materials and methods

### Clinical samples

Plasma and placental villous tissues were collected from 60 pregnant women with GDM and 60 healthy control pregnant women at Shengjing Hospital from January 2016 to June 2019. Plasma samples were collected from all pregnant women. Placental villous tissues were obtained immediately after delivery. Also, we obtained placenta homogenate according to the following steps: we separated 5 g placental villous tissues; the samples were macerated with saline solution and centrifuged at 500g for 20 min, and then the supernatant was stored in 5 ml aliquots at 20°C. The exclusion criteria were as follows: multiple pregnancies, history of type 2 diabetes, alcohol abuse, hypertension, smoking, and assisted reproductive technology treatment. GDM was diagnosed based on 75 g oral glucose tolerance test (OGTT). GDM was diagnosed when at least one of the following conditions was met: FPG ≥ 5.1 mmol/L, one-hour OGTT ≥ 10.0 mmol/L, or two-hour OGTT ≥ 8.5 mmol/L. Written informed consent was obtained from all participants. The Ethics Committee of Shengjing Hospital approved this research.

### Analysis of circ_0001578 in placenta-derived exosomes and placental villous tissues

We collected plasma samples for exosomes isolation at the 12th, 24th, and 36th week of pregnancy, separately. The placenta-derived exosomes were extracted using Exosome Isolation Reagent (Geenseed, Guangzhou, China). The plasma was diluted with an equal volume of PBS (pH 7.4) and centrifuged at 3000 × *g* for 20 min at 4°C (Sorvall High Speed Microcentrifuge, fixed rotor; Thermo Fisher Scientific, Asheville, NC, USA). Then, 3000 μg of the supernatant fluid was filtered through a 0.22-mm filter (Steritop; Millipore). PEG 6000 (final concentration 8%) was added to the filtrate and mixed. The filtrate was incubated at 4°C overnight and centrifuged at 10,000 × *g* for 60 min at 4°C (Sorvall High Speed Microcentrifuge, fixed rotor). The pellet (10,000 μg) was suspended in sucrose solution (0.25 mol/L). The 100,000 g pellet was resuspended in 500 ml of PBS and layered on the top of a discontinuous sucrose gradient containing 40% (weight-to-volume ratio [w/v]), 20% (w/v), 10% (w/v), and 5% (w/v) sucrose solution and centrifuged at 100,000 × *g* for 5 h. Solutions were made by diluting a stock solution of OptiPrep™ (60% [w/v] aqueous sucrose solution from Sigma-Aldrich). An exosome-containing fraction (density, 1.08 to 1.10 g/ml) was collected, diluted with PBS, and centrifuged at 100,000 × *g* for 2 h at 4°C. Finally, the pellet containing the enriched exosome population was resuspended in 50 ml of PBS. Exosomes were characterized by measuring the size distribution and morphology by Western blotting and transmission electron microscopy, respectively.

TRIzol (Invitrogen, Waltham, MA, USA) was used to isolate total RNAs. Additionally, 100 mg of frozen placental villous tissue was crushed and treated with TRIzol (Invitrogen) to isolate total RNAs. Prime Script RT Reagent (Takara, Kusatsu, China) was used to synthesize cDNAs. The ABI PRISM7500 System (Applied Biosystems, Waltham, MA, USA) was used for qRT-PCR. The reaction conditions were 95°C for 10 min, 95°C for 10 s, and 59°C for 60 s for 40 cycles, and 70°C for 30 s. *GAPDH* was used as endogenous control. Then, 2 μg of total RNAs was treated with 6 U of RNase R (Thermo, Waltham, MA, USA), and the resulting RNAs were purified using the RNeasy Min Elute cleaning kit (Qiagen, Shanghai, China). Relative expression levels were determined by the 2^−ΔΔCt^ method. Primers for circRNA_0001578 and *GAPDH* for qRT-PCR were as follows: circRNA_0001578, 5′-CCCTCAGGGAGCCTCCTG-3′ (sense), 5′-TAGCAGTCGCGGACGGTG-3′ (antisense); *GAPDH*, 5′-GAAGGTGAAGGTCGGAGTC-3′ (sense), 5′-GAAGATGGTGATGGGATTTC-3′ (antisense).

### Cell transfection

The HTR-8/SVneo cell line (ATCC) was derived from embryonic trophoblast villi of a human first-trimester placenta. HTR-8/SVneo cells are epithelial cell-like. Trophoblast HTR-8/SVneo cells were maintained in an RPMI 1640 medium, supplemented with 10% fetal bovine serum and antibiotics and cultured in humidified atmosphere with 5% CO_2_, at 37°C. Upon seeding, small interfering RNAs (siRNAs; Thermo), targeting circ_0001578, and their negative controls (si-NC; Thermo) were used to transfect HTR-8/SVneo cells. The protocol for cell transfection in a 6-well plate is as follows: plate 2.5×10^5^ cells per well in 2 ml of a complete growth medium 12 h prior to transfection; wash with 1×PBS and add 2 ml of the RPMI 1640 medium; prepare transfection complexes by mixing 40 μl of a serum-free medium, 6 μl of Lipofectamine 3000, and 0.67 ug of specific siRNA or si-NC; incubate transfection complexes at room temperature for 20 min; add prepared transfection complexes to 2 ml of the RPMI 1640 medium; and incubate cells at 37°C in a humidified CO_2_ incubator. The cells were treated for 24 h. As described above, we also cotransfected HTR-8/SVneo cells with si-NF-κB (Thermo) and si-circ_0001578. Also, we treated HTR-8/SVneo cells with si-circ_0001578 and 15 nM SP600125 (an inhibitor of JNKs, Thermo).

### CCK8 assay

The Cell Counting Kit-8 Assay (Glpbio, Montclair, CA, USA) was used to determine cell survival. Transfected cells were seeded and incubated in 96-well plates, and the density was about 5,000 cells per well. CCK-8 reagent (10 ml) was added at the time points of 0, 24, 48, or 72 h. Viable cells were counted by detecting absorbance at 450 nm using a microtiter plate reader (Bio-Tek, Winooski, VT, USA). The optical absorbance reflects the cell survival conditions. The experiments were repeated at least three times with three replicates each.

### Colony formation assay

In this assay, transfected HTR-8/SVneo cells were plated at a density of 2,000 cells/well in six-well plates and cultured. Two weeks later, the medium was removed and the plates were washed three times with phosphate-buffered saline (PBS). The cells were fixed with methanol for 30 min and then stained with hematoxylin for 20 min. The plates were dried with a blower to ensure that high-quality images were obtained. The colonies were defined as >50 cells/colony. The experiments were repeated at least three times with three replicates each.

### Wound healing assay

Transfected HTR-8/SVneo cells were seeded at a density of 10^5^ cells/well in six-well plates and incubated overnight. A sterile 200-μl micropipette tip was used to scratch the cell monolayer. After washing three times with phosphate-buffered saline (PBS), a culture medium was added. Representative images of cell migration were captured by photographing 10 high-power fields at 0 and 24 h after injury. Image Pro Plus software was used to determine the cell migration distance. The experiments were repeated at least three times with three replicates each.

### Transwell assay

Transwell (24-well plate) with 0.8-mm aperture (Corning, USA) was applied for invasion assay. Transfected HTR-8/SVneo cells were starved overnight in a medium without fetal bovine serum, then 10^5^ cells resuspended in a 0.1 ml serum-free medium were added to the apical chamber, and RPMI 1640 containing 10% fetal bovine serum was added to the basolateral chamber as a chemoattractant. The cells were cultured for 24 h, according to the instructions of the Transwell chamber, and then the cells of the apical chamber were cleaned. The chamber was then rinsed with PBS and immersed with precooled methanol for 30 min. Cells transferred to the basolateral chamber were fixed and stained with 0.1% crystal violet (Topscience, Shanghai, China) for 10 min. Six visual fields were selected and the cells were observed, photographed, and counted under an inverted microscope (Olympus, Tokyo, Japan). The experiments were repeated at least three times with three replicates each.

### Western blotting

RIPA buffer was used to lyse HTR-8/SVneo cells for protein extraction. Total protein was quantified by the Bradford method. Twenty micrograms of protein, diluted in NuPAGE sample buffer, was denatured at 95°C for 5 min. Equal amounts of protein samples were separated by 8% SDS-PAGE (100v, 100 min, DETAI Science, Ningbo, China). Following electrophoresis, the proteins were blotted onto PVDF membranes at 100 V for 90 min (Millipore, Billerica, MA, USA). Then, 5% evaporated milk was used to block the membranes at room temperature for 1 h. Membranes were then incubated with primary antibodies at 4°C overnight. The primary antibodies were anti-JNK (1:500; #ab124956, Abcam, Cambridge, MA, USA), anti-NF-κB (1:500; #610868, BD, Franklin Lakes, NJ, USA), and anti-β-actin (1:1000; #ab8226, Abcam). After extensive washing, the membranes were incubated with the secondary antibody (1:5000, #31460, and #31430; Thermo Fisher) at room temperature for 2 h. After several washing steps, the membranes were developed with the Electro-Chemi-Luminescence (ECL; Amersham Biosciences, UK), and the protein bands were documented with the GelDoc Imaging System. Image J software was used to quantify the protein bands relative to β-actin. The experiments were repeated at least three times.

### Apoptosis assays

Apoptosis assays were performed using 7-AAD/Annexin V-APCApoptosis Detection Kit (Hanyi Biosciences, China). In brief, transfectedHTR-8/SVneo cells were harvested and washed twice with PBS. Annexin V binding solution (400 μl) suspended the cells at a concentration of about 2×10^6^ cells/ml. Annexin V-APC staining solution (5 μl) was added into the 100 μl cell suspension and then incubated at 4°C for10 minutes. 7-AAD (5 μl) was then mixed with the solution and incubated for 3 minutes at 4°C. After being diluted with 400 μl PBS, a flow cytometry assay was performed using an LSR II flow cytometer (BD Biosciences, USA). FlowJo was used to evaluate the results. 7-AAD and Annexin V double-positive cells were defined as apoptotic cells.

### ELISA

ELISA kits for IL-1 (#ab235646; Abcam), IL-6 (#ab178013; Abcam), IL-8 (#ab185986; Abcam), TNF-α (#ab181421; Abcam), and CRP (#ab260058; Abcam) were used to measure the concentration of inflammatory markers in HTR-8/SVneo cell culture media of cells transfected with si-NC or targeted si-RNA and placenta homogenate of GDM patients and control. We also detected the above markers in HTR-8/SVneo cell culture media of cells treated with si-circ_0001578 and Pyrrolidinedithiocarbamate ammonium (PDTC) or SP600125. ELISA tests were used according to the manufacturer’s instructions. Briefly, 96-well plates were coated with antibodies at 4°C overnight, loaded with 100 μl of standard or condition media, and incubated at room temperature for 2 h. Samples were sequentially incubated with Detection Reagent A and B for 1 h at room temperature. Then, the antigen–antibody complex was determined by the addition of TMB substrate and observation at an OD of 450 nm. The experiments were repeated at least three times.

### Colorimetric caspase-3 assays

Caspase-3 activity was determined by Caspase-3 Assay Kit (Colorimetric). Cells were washed with PBS, resuspended in 50 ml cell lysis buffer, and incubated on ice for 10 min. The cell lysates were pelleted, and the supernatants were transferred to microcentrifuge tubes. The 50 ml cell lysates and 50 ml of the reaction buffer were added to the microplate well. Subsequently, 5 ml of the 4 mM DEVD-pNA substrate was added and incubated at 37°C for 2 h. A microplate reader (Bio-Tek) was used to measure absorbance at 450 mm.

### Statistical analysis

SPSS 22.0 (SPSS Inc., Chicago, IL, USA) was used to perform two-tailed Student’s *t*-tests. A receiver operating characteristic (ROC) curve analysis was used to determine the diagnostic value of circ_0001578 for GDM. P < 0.05 was considered statistically significant. All experiments were repeated three times.

## Results

### Circ_0001578 is downregulated in placental villous tissues of pregnant women with GMD

**Figure 1:**
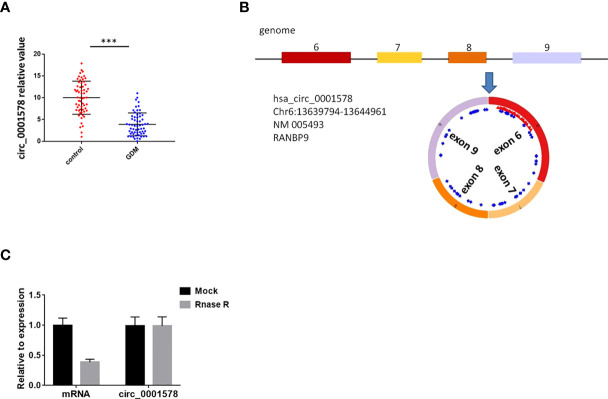
CircRNAcirc_0001578 was downregulated in the placental villous tissues of pregnant women with GDM. **(A)** Relative expression levels of circ_0001578 in the placental villous tissues of pregnant women with or without GMD (3.88 vs.10, P < 0.001). **(B)** Circ_0001578 originates from splicing exons 6, 7, 8, and 9 of *RANBP9.* **(C)** Circ_0001578 was resistant to RNase R digestion, unlike linear *RANBP9* mRNA ***P<0.001.

Wang et al. have reported that 43 circRNAs are downregulated in the placental villous tissues of patients with GMD based on an RNA-seq analysis (31). We validated five circRNAs (circ_0001578, circ_0006260, circ_0003218, circ_0000139, and circ_0002466) in the placental villous tissues of 30 pregnant women with GDM and 30 healthy control pregnant women by qRT-PCR (**Supplemental Figure 1**). These five circRNAs have the most significant P value in Wang’s RNA-seq analysis. Consistent with the results of Wang’s RNA-seq analysis, these circRNAs were significantly downregulated in placental villous tissues of patients with GMD. Among the five circRNAs, circ_0001578 had the most significant differential expression. Therefore, we selected circ_0001578. Furthermore, we validated circ_0001578 expression in the placental villous tissues of 60 pregnant women with GDM and 60 healthy control pregnant women by qRT-PCR. We found that circ_0001578 was significantly downregulated in placental villous tissues of patients with GDM (**Figure 1A**). Circ_0001578 originates from the *RANBP9* gene. It is 598 bp in length consisting of splicing exons 6, 7, 8, and 9 of *RANBP9* (**Figure 1B**). Furthermore, compared with linear *RANBP9* mRNA, circ_0001578 had strong resistance to RNase R treatment, indicating that circ_0001578 was a circular RNA (**Figure 1C**). Linear *RANBP9* mRNA expression in the placental villous tissues of 60 pregnant women with GDM and 60 healthy control pregnant women were also detected by qRT-PCR. We found no statistical difference (**Supplemental Figure 2A**).

### Diagnostic value of circ_0001578 in GDM

**Figure 2:**
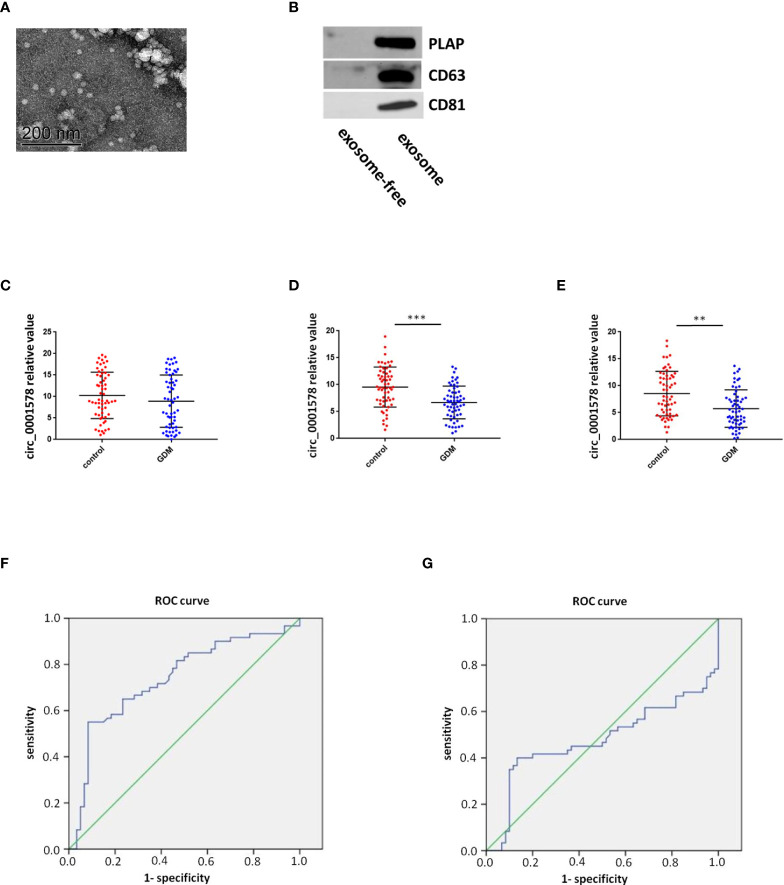
Diagnostic value of placenta-derived exosome circ_0001578 in GDM. **(A)** Identification of placenta-derived exosomes by TEM and Western blotting. **(B)** Identification of placenta-derived exosomes by Western blotting. **(C)** Placenta-derived exosome circ_0001578 expression in GDM and control groups in the first trimester (8.93 vs. 10.00, P = 0.21). **(D)** Placenta-derived exosome circ_0001578 expression in GDM and control groups in the second trimester (6.19 vs. 9.56, P < 0.05). **(E)** ROC curve analyses of the diagnostic value of placenta-derived exosomecirc_0001578 in the second trimester. **(F)** Placenta-derived exosome circ_0001578 expression in GDM and control groups in the third trimester (5.75 vs. 8.61, P < 0.05). **(E)** Placenta-derived exosome circ_0001578 expression in GDM and control groups in the third trimester (5.75 vs. 8.61, P < 0.05). **(F)** ROC curve analyses of the diagnostic value of placenta-derived exosomecirc_0001578 in the second trimester. **(G)** ROC curve analysis of placenta-derived exosome circ_0001578 in the third trimester.

The expression of circ_0001578 in placenta-derived exosomes in maternal circulation from 60 patients with GDM and 60 healthy control pregnant women was determined with RT-qPCR in the first, second, and third trimesters (12th, 24th, and 36th weeks). First, placenta-derived exosomes were identified by transmission electron microscopy (**Figure 2A**) and Western blotting (**Figure 2B**). CD63 and CD81 are exosome markers (36) and PLAP is a specific marker of placental exosomes (37). The difference in placenta-derived exosome circ_0001578 expression between the GDM and control groups was not statistically significant in the first trimester (**Figure 2C**). Compared with levels in the control group, placenta-derived exosome circ_0001578 expression levels were significantly lower in the GDM group in the second trimester (P < 0.001, **Figure 2D**). Furthermore, the diagnostic value of placenta-derived exosome circ_0001578 expression was evaluated by an ROC curve analysis (**Figure 2F**). The AUC (area under the curve) was 0.738 (P < 0.05). In the third trimester, placenta-derived exosome circ_0001578 expression was significantly lower in the GDM group than in the control group (**Figure 2E**). An ROC curve analysis (**Figure 2G**) revealed an AUC value of 0.478 (P = 0.091). The predictive value of placenta-derived exosome circ_0001578 expression in the second trimester was better than that in the third trimester. The diagnostic sensitivity (67.5%) and specificity (73.4%) were highest when the cutoff relative value was 6.93, suggesting the best predictive performance at this level. These results indicated that placenta-derived exosome circ_0001578 expression in the second trimester has the potential to serve as an earlier marker of GDM.

### Downregulation of circ_0001578 inhibits proliferation and migration and induces apoptosis in trophoblast cells

**Figure 3:**
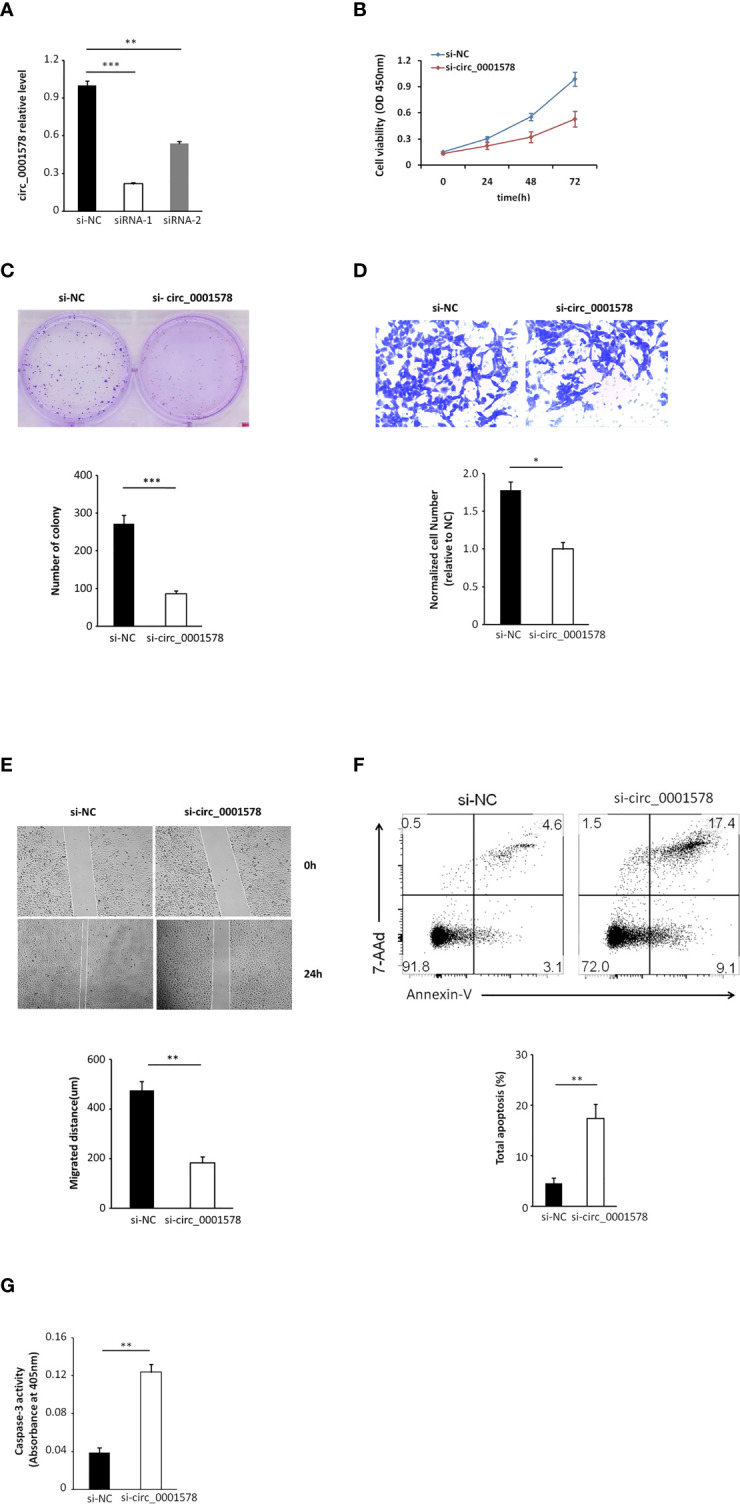
Downregulation of circ_0001578 inhibits proliferation and migration and induces apoptosis in trophoblast cells. **(A)** The interference efficiency of si-circRNAs was determined by qRT-PCR (siRNA-1, P < 0.001; siRNA-2, P < 0.01). **(B)** Viability of HTR-8/SVneo cells after si-circRNA or si-NC treatment at 48 and 72 h after transfection (P < 0.05 at 48 h, P < 0.01 at 72 h). **(C)** Clone formation assay of HTR-8/SVneo cells transfected with si-circRNA or si-NC (P < 0.0001). **(D)** Migration ability of HTR-8/SVneo cells transfected with si-circRNA or si-NC was determined by a transwell assay (P < 0.05). **(E)** Migration ability of HTR-8/SVneo cells transfected with si-NC or si-circRNA was determined by a wound healing assay (P < 0.01). **(F)** Apoptosis of HTR-8/SVneo cells transfected with si-NC or si-circRNA was determined by Annexin-V staining (P < 0.01). **(G)** Apoptosis of HTR-8/SVneo cells transfected with si-NC or si-circRNA was determined by caspase-3 assays (P < 0.01) *P<0.05; **P<0.01; ***P<0.001.

We transfected HTR-8/SVneo trophoblasts with siRNAs targeting circ_0001578 (si-circRNAs) or si-NC. HTR-8/SVneo cells transfected with negative control siRNA were defined as negative controls. The interference efficiency of si-circRNAs was determined by qRT-PCR (**Figure 3A**). Between the two si-circRNAs evaluated, si-circRNA-1 showed a better interference efficiency and was used in further experiments. Downregulation of circ_0001578 did not affect RANBP9 (the host gene of circ_0001578) expression (**Supplemental Figure 2B**). Cell viability was significantly lower following si-circRNA interference than in the control group at 48 and 72 h after transfection (**Figure 3B**). Similarly, the si-circRNA group showed significantly fewer clones relative to the si-NC group in clone formation assays (**Figure 3C**). A transwell migration assay indicated that the downregulation of circ_0001578 decreased cell migration (**Figure 3D**). A wound healing assay showed similar results. The migration distance in the si-circRNA group was significantly lower than that in the si-NC group (**Figure 3E**). To explore the effect of circ_0001578 on HTR-8/SVneo apoptosis, Annexin-V staining was conducted. The downregulation of circ_0001578 significantly promoted cell apoptosis (**Figure 3F**). Colorimetric caspase-3 assays revealed similar results (**Figure 3G**). Collectively, these data indicated that the downregulation of circ_0001578 inhibits the proliferation and migration of trophoblasts and accelerates apoptosis *in vitro*.

### Downregulation of circ_0001578 induces inflammation *via* NF-κB and JNK pathways

**Figure 4:**
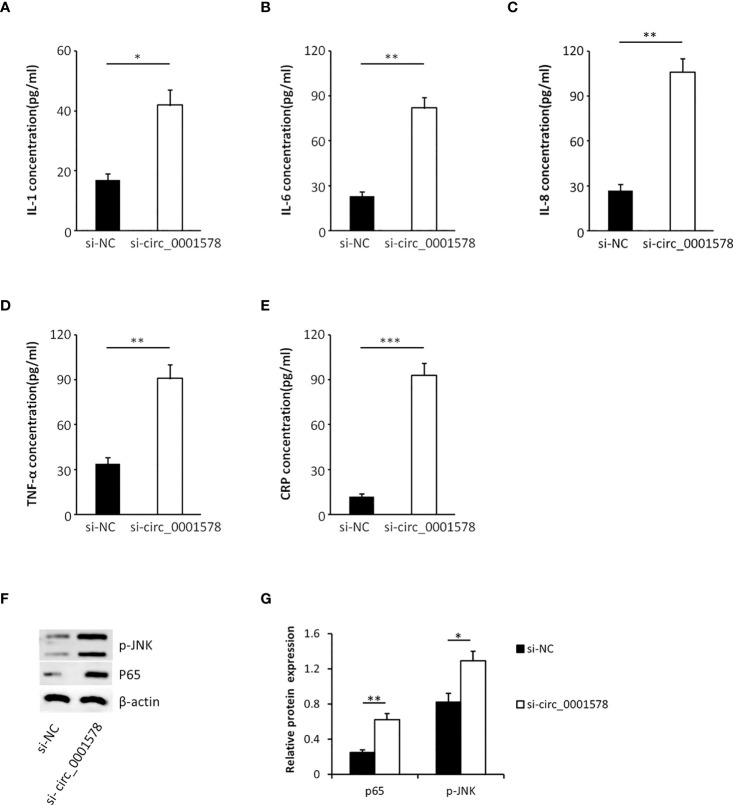
Downregulation of circ_0001578 induces inflammation in trophoblasts. **(A)** IL-1 levels were significantly higher in the culture medium treated with si-circRNA than in the si-NC group (P < 0.05). **(B)** IL-6 levels were significantly high in the culture medium treated with si-circRNA than in the si-NC group (P < 0.01). **(C)** IL-8 levels were significantly higher in the culture medium treated with si-circRNA than in the si-NC group (P < 0.01). **(D)** TNF-α levels were significantly higher in the culture medium treated with si-circRNA than in the si-NC group (P < 0.01). **(E)** CRP levels were significantly higher in the culture medium treated with si-circRNA than in the si-NC group (P < 0.001). **(F)** Expression levels of p-JNK and nuclear P65 followed by circ_0001578 knockdown detected by Western blotting. **(G)** Semiquantitative analysis of protein expression *P<0.05; **P<0.01; ***P<0.001.

In the pathogenesis of GDM, inflammation plays an important role and inflammatory mediators have been identified as risk factors for the progression of GDM (38). To determine the effect of circ_0001578 downregulation on inflammatory mediators, we used ELISA to detect inflammatory mediators in the HTR-8/SVneo cell culture medium. Levels of IL-1 (**Figure 4A**), IL-6 (**Figure 4B**), IL-8 (**Figure 4C**), TNF-α (**Figure 4D**), and CRP (**Figure 4E**) were significantly higher in the si-circRNA-treated culture medium than in the si-NC group. We concluded that the downregulation of circ_0001578 may induce inflammation in the placenta, which may be related to GDM.

To explore the underlying mechanism by which the downregulation of circ_0001578 induces inflammation, we analyzed the expression levels of nuclear NF-κB (P65 subunit) and phospho-JNK by Western blot. Significantly increased expression of nuclear P65 and p-JNK following circ_0001578 knockdown (**Figures 4F, G**) suggests that downregulation of circ_0001578 induces inflammation *via* the NF-κB and JNK pathways.

### Inhibition of NF-κB and JNK restore inflammation induced by aberrant circ_0001578 expression

**Figure 5:**
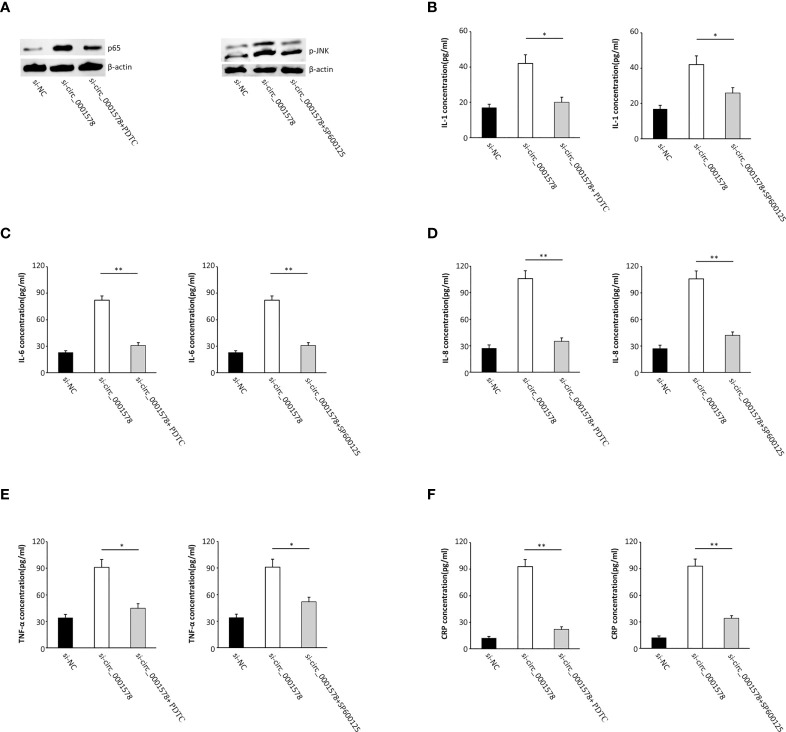
Inhibition of NF-κB and JNK pathways restores the inflammation of trophoblasts induced by the aberrant expression of circ_0001578. **(A)** PDTC reversed the nuclear translocation of NF-κB; SP600125 inhibited JNK activation. **(B)** IL-1 levels were reversed by PDTC or SP600125 treatment compared with levels in the single si-circ_0001578 transfection group (P < 0.05). **(C)** IL-6 levels were reversed by PDTC or SP600125 treatment compared with levels in the single si-circ_0001578 transfection group (P < 0.01). **(D)** IL-8 levels were reversed by PDTC or SP600125 treatment compared with levels in the single si-circ_0001578 transfection group (P < 0.01). **(E)** TNF-α levels were reversed by PDTC or SP600125 treatment compared with levels in the single si-circ_0001578 transfection group (P < 0.05). **(F)** CRP levels were reversed by PDTC or SP600125 treatment compared with levels in the single si- circ_0001578 transfection group (P < 0.01) *P<0.05; **P<0.01.

Cells were transfected with si-circ_0001578 and treated with pyrrolidinedithiocarbamate ammonium (PDTC, a NF-κB activity inhibitor) to reverse the nuclear translocation of NF-κB. Additionally, SP600125, a JNK signaling antagonist (39), was used to treat cells together with si-circ_0001578 to inhibit JNK activation (**Figure 5A**). We used ELISA to detect inflammatory mediators in the HTR-8/SVneo cell culture medium. The si-circ_0001578-induced changes in the levels of IL-1 (**Figure 5B**), IL-6 (**Figure 5C**), IL-8 (**Figure 5D**), TNF-α (**Figure 5E**), and CRP (**Figure 5F**) were reversed by PDTC or SP600125 treatment. These results revealed that the inhibition of NF-κB and JNK pathways restores the inflammation of trophoblasts induced by the aberrant expression of circ_0001578. We concluded that the downregulation of circ_0001578 induces inflammation of trophoblast cells *via* NF-κB and JNK pathways.

### Inflammation in placental villous tissues of pregnant women with GMD

**Figure 6:**
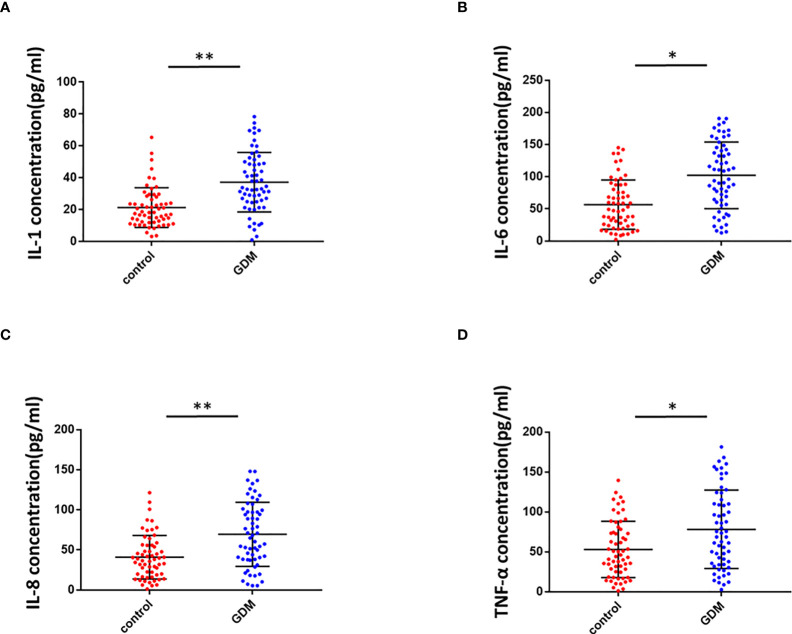
Inflammation in the placental villous tissues of pregnant women with GMD. **(A)** IL-1 levels were significantly higher in the placental villous tissues of pregnant women with GMD (P < 0.01). **(B)** IL-6 levels were significantly higher in the placental villous tissues of pregnant women with GMD (P < 0.05). **(C)** IL-8 levels were significantly higher in the placental villous tissues of pregnant women with GMD (P < 0.01). **(D)** TNF-α levels were significantly higher in the placental villous tissues of pregnant women with GMD (P < 0.05). *P<0.05; **P<0.01.

To determine the inflammation in the placental villous tissues of pregnant women with GMD, we used ELISA to detect inflammatory mediators in the placenta homogenate of the same 60 pregnant women with GDM and 60 healthy control pregnant women. Levels of IL-1 (**Figure 6A**), IL-6 (**Figure 6B**), IL-8 (**Figure 6C**), and TNF-α (**Figure 6D**) were significantly higher in the GDM group than the control. We concluded that inflammation of placenta occurred in GDM patients.

## Discussion

In this study, we extracted previous RNA-seq results (31) and validated five circRNAs. We found the significant downregulation of circ_0001578 in the placental villous tissues of pregnant women with GMD by qRT-PCR. Circ_0001578 originates from the *RANBP9* gene. It is 598 bp in length consisting of splicing exons 6, 7, 8, and 9 of *RANBP9*. Linear *RANBP9* mRNA expression had no statistical difference between GDM and control pregnant women. There is no literature report on its role in GDM. The early detection of GDM is very important, and therefore, we investigated the diagnostic value of placenta-derived exosome circ_0001578 expression by transmission electron microscopy and marker expression. We found that placenta-derived exosome circ_0001578 expression in the second trimester has the potential to serve as an early marker of GDM. Furthermore, our *in vitro* experiments showed that the downregulation of circ_0001578 inhibits migration and proliferation and induces apoptosis in trophoblasts. Placental trophoblasts are very active during pregnancy. The maintenance of placental function depends on the normal migration ability of trophoblasts (21). Dysfunction of trophoblasts can lead to the dysfunction of the placenta, inducing inflammation in placental villous tissues. GDM involves the abnormal function and inflammation of placental villous tissues (34). Consistent with the important role of inflammation in the pathogenesis of GDM (38), we found that downregulation of circ_0001578 induces significantly increased levels of IL-1, IL-6, IL-8, TNF-α, and CRP in trophoblasts. IL-1, IL-6, and IL-8 are involved in the development of GDM by acting as inflammatory regulators. They are involved in the inflammation of the placenta in GDM (38, 40). The level of TNF-α is elevated in the adipose tissues of obese individuals, and TNF-α decreases insulin sensitivity. Weight loss is also associated with the decreased TNF-α level and restored insulin sensitivity. During pregnancy, TNF-α secreted by the placenta causes inflammatory cell aggregation and impairs insulin resistance. TNF-α is associated with the development of GDM (41). CRP levels are low in nonpregnant and healthy individuals. CRP also has as a crucial role in GDM (42). Our results further indicate that circ_0001578 acts as an inflammatory regulator. With respect to the regulatory mechanism underlying its effects on inflammation, we detected the aberrant nuclear translocation of NF-κB and activation of the JNK pathway. NF-κB is a prototypical proinflammatory signaling pathway. Nuclear translocation of NF-κB activates the pathway (43). JNKs are a family of stress-activated serine threonine protein kinases of the MAPK. The JNK family includes JNK1, JNK2, and JNK3. JNK1 and JNK2 are widely expressed in various tissue types, while JNK3 is specifically expressed in the cardiac smooth muscle and central nervous system. Additionally, phosphorylation of JNK1 and JNK2 is crucial for insulin resistance and diabetes (44). In this study, nuclear translocation of NF-κB and phosphorylation of JNK were significantly elevated following the downregulation of circ_0001578 in HTR-8/SVneo cells, which may induce inflammation in the placenta. Furthermore, the inhibition of nuclear translocation of NF-κB and p-JNK attenuated the circ_0001578-induced changes in IL-1, IL-6, IL-8, TNF-α, and CRP levels. Also, we found that inflammatory makers (IL-1, IL-6, IL-8, and TNF-α) were significantly higher in the placental villous tissues of GDM.

The observed effects of the downregulation of circ_0001578 on proliferation, migration, and apoptosis in trophoblasts may explain chronic inflammation in the placenta, which can drive insulin resistance and lead to decreased insulin sensitivity. These effects may be caused by the activation of NF-κB and JNKs pathways, combined with increased expression levels of IL-1, IL-6, IL-8, TNF-α, and CRP.

Collectively, the downregulation of circ_0001578 may promote GDM by inducing chronic inflammation of the placenta *via* the NF-κB and JNK pathways. Placenta-derived exosome circ_0001578 expression in the second trimester has the potential to serve as an early marker of GDM.

## Data availability statement

The original contributions presented in the study are included in the article/**Supplementary Material**. Further inquiries can be directed to the corresponding authors.

## Ethics statement

The studies involving human participants were reviewed and approved by the Ethics Committee of Shengjing Hospital. The patients/participants provided their written informed consent to participate in this study.

## Author contributions

XZ contributed to statistical analyses and writing the manuscript, WZ contributed to the experiments, LL contributed to critical discussion. All authors contributed to the article and approved the submitted version.

## Funding

This work was supported by the Project of Department of Education of Liaoning Province (Grant no. QNZR2020017) and China Postdoctoral Science Foundation (Grant no. 2022MD713825) and 345 Talent Project of Shengjing Hospital of China Medical University and Project of Shenyang Science and Technology Bureau (Grant no. RC210316)

## Conflict of interest

The authors declare that the research was conducted in the absence of any commercial or financial relationships that could be construed as a potential conflict of interest.

## Publisher’s note

All claims expressed in this article are solely those of the authors and do not necessarily represent those of their affiliated organizations, or those of the publisher, the editors and the reviewers. Any product that may be evaluated in this article, or claim that may be made by its manufacturer, is not guaranteed or endorsed by the publisher.

## Supplementary material

Supplementary Figure 1 Validation of circ_0001578 **(A)**, circ_0006260 **(B)**, circ_0003218 **(C)**, circ_0000139 **(D)**, and circ_0002466 **(E)** in placental villous tissues of 30 pregnant women with GDM and 30 healthy control pregnant women by qRT-PCR.

Supplementary Figure 2
**(A)** Linear RANBP9 mRNA were detected in placental villous tissues of 60 pregnant women with GDM and 60 healthy control pregnant women by qRT-PCR. **(B)** Downregulation of circ_0001578 did not affect RANBP9 (the host gene of circ_0001578) expression.
