# Supplementary figures and images for: Downregulationof circ_0001578 promotes gestational diabetes mellitus by inducing placental inflammation via the NF-κB and JNKs pathways
Source: Front Endocrinol (Lausanne). 2022 Oct 3;13:657802. doi: 10.3389/fendo.2022.657802 (PMC9573949; doi:10.3389/fendo.2022.657802)

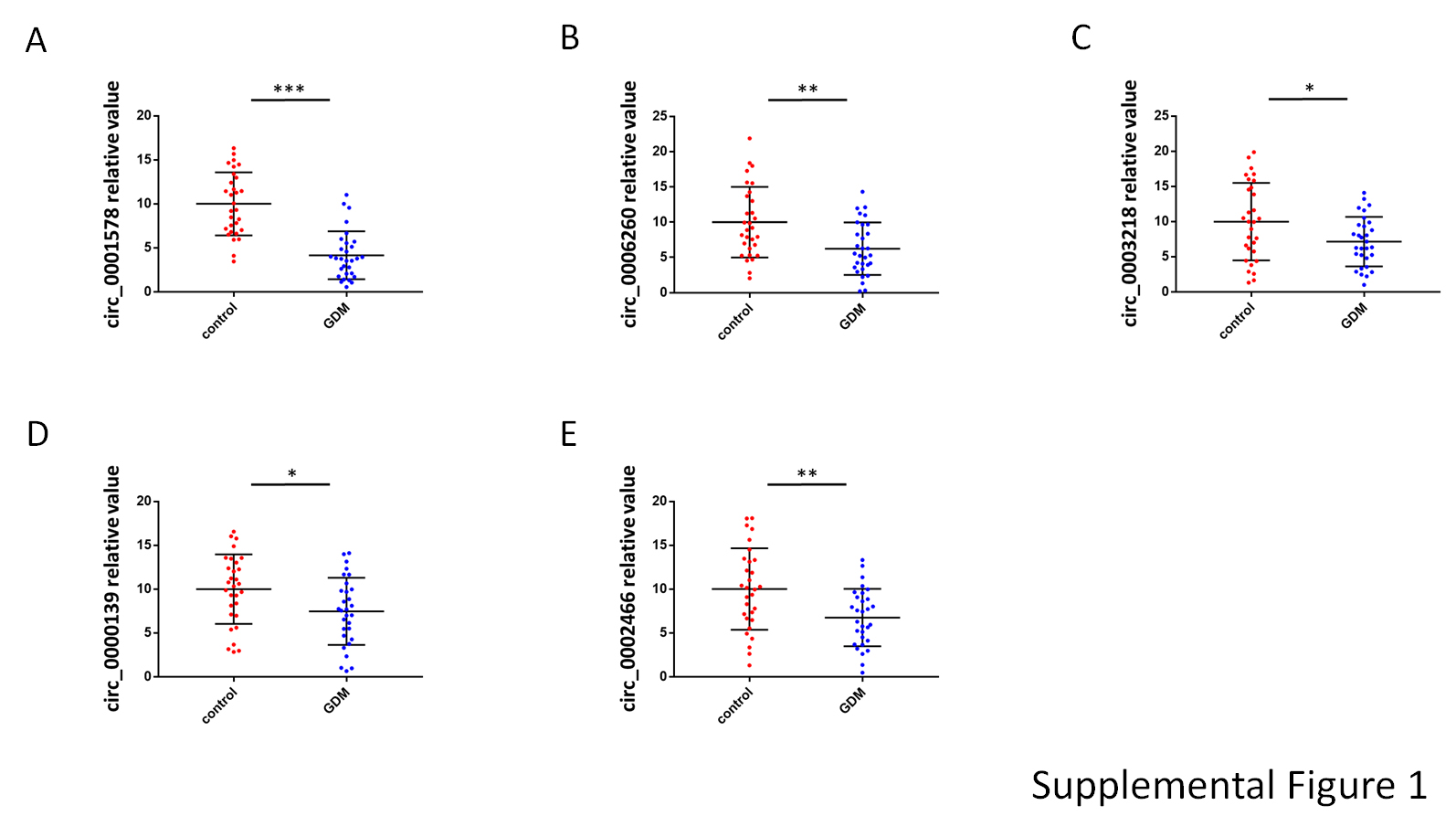

Supplement: Supplementary Figure 1 — Validation of circ_0001578 (A), circ_0006260 (B), circ_0003218 (C), circ_0000139 (D), and circ_0002466 (E) in placental villous tissues of 30 pregnant women with GDM and 30 healthy control pregnant women by qRT-PCR. [file Image_1.jpeg]

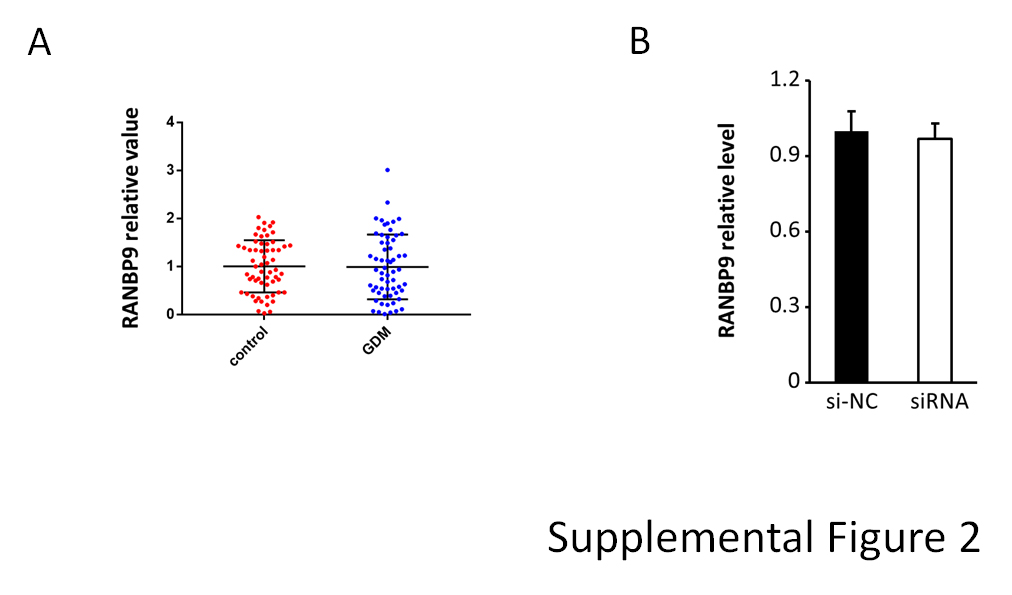

Supplement: Supplementary Figure 2 — (A) Linear RANBP9 mRNA were detected in placental villous tissues of 60 pregnant women with GDM and 60 healthy control pregnant women by qRT-PCR. (B) Downregulation of circ_0001578 did not affect RANBP9 (the host gene of circ_0001578) expression. [file Image_2.jpeg]
